# Monocyte-derived dendritic cells identified as booster of T follicular helper cell differentiation

**DOI:** 10.1002/emmm.201404015

**Published:** 2014-05-06

**Authors:** Simon Fillatreau

**Affiliations:** Deutsches Rheuma-Forschungszentrum, a Leibniz InstituteBerlin, Germany

## Abstract

Adjuvants play an essential role in the induction of acquired immunity upon vaccination with protein antigen. In this issue of *EMBO Molecular Medicine*, a classical type of adjuvant made of DNA oligonucleotide containing CpG motifs, which has already been used in humans, is shown to boost humoral immunity primarily by acting on monocyte-derived dendritic cells. This study provides novel insight on the mode of action of adjuvant targeting Toll-like receptors.

The humoral component of acquired immunity was discovered by Emil von Behring and Shibasaburo Kitasato about a century ago during their work on serum therapy against diphtheria. Their findings were rapidly exploited, and serotherapy saved thousands of lives from infectious diseases. Acquired humoral immunity is also the primary mechanism underlying the protective effect afforded by current vaccines against infectious diseases. The persistence of acquired humoral immunity after an immune challenge can be remarkably long lasting. Levels of specific antibodies toward tetanus or diphtheria displayed half-lives of 11–19 years and up to 500–3,000 years for mumps and measles (Amanna *et al*, 2007). The maintenance of acquired humoral immunity after vaccination or resolution of an infection results from the formation of long-lived memory B cells and plasma cells.

After an immune challenge, activated B cells can differentiate into memory B cells and long-lived plasma cells in the germinal centers that develop within the B-cell follicles under the command of T-cell-dependent processes. Indeed, CD4^+^ T cells provide cognate B cells with the helper signals required for their initiation of the germinal center reaction and subsequently for their survival and final differentiation. T-cell help is considered a major limiting factor for the germinal center response. A key goal in vaccinology is therefore to characterize the T helper cell driving the germinal center reaction and to identify the mechanisms underlying their differentiation. A major breakthrough was the identification of T follicular helper (T_FH_) cells, a specific CD4^+^ T-cell subset that characteristically expresses the master transcription factor Bcl-6, and the chemokine receptor CXCR5, which allows their migration into B-cell follicles where the chemokine CXCL13 is produced by follicular dendritic cells (Crotty, 2011). Evidence is accumulating that T_FH_ cells can become memory cells (Weber *et al*, 2012). In the sole absence of these T cells, germinal centers, memory B cells, and long-lived plasma cells fail to develop efficiently. The study by Chakarov and Fazilleau published in this issue of *EMBO Molecular Medicine* provides important new findings on the signals and cells that can promote T_FH_-cell differentiation (Chakarov & Fazilleau, 2014).

CpG increased the formation of antigen-reactive T_FH_ cells in various immunization regimens

The current trend in vaccination relies on the usage of highly purified antigens combined with adjuvants that stimulate optimal adaptive immune responses. Toll-like receptor (TLR) agonists constitute a particularly promising class of adjuvants because they can stimulate both innate and adaptive immunity and are thought to promote potent long-term protection. Consistent with this view, one of the most successful vaccines, the live attenuated yellow fever vaccine 17D, has a remarkable capacity to stimulate multiple TLRs. The TLR9 agonists comprising CpG-containing DNA oligonucleotides have been the focus of intense interest for the production of better vaccines for humans. Addition of a CpG oligonucleotide (called CpG 7909 or CpG 2006) to a hepatitis B vaccine induced a long-lasting protective antibody response in HIV-infected subjects who had previously failed to respond to the same vaccine in the absence of such adjuvant (Cooper *et al*, 2005). The study of Chakarov and Fazilleau addresses how CpG improves the development of acquired humoral immunity toward protein antigens. Following the observation that CpG increased the formation of antigen-reactive T_FH_ cells in various immunization regimens, they examined which cell type was responsible for this effect. CpG oligonucleotides exist in distinct flavors that differ by their capacity to preferentially stimulate B cells versus myeloid cells. The CpG oligonucleotide used in this study (and in human vaccination trials) preferentially activates B cells (and few specific dendritic cell (DC) subsets), suggesting that it might promote T_FH_-cell formation via intrinsic signaling in B cells, which express TLR9 and can contribute to the formation of T_FH_ cells. However, Chakarov and Fazilleau found comparable formation of antigen-specific T_FH_ cells in wild-type and B-cell-deficient mice, which also displayed similar T_FH_-cell number increase when CpG was added to the immunization cocktail. These findings confirm previous observation that T_FH_-cell differentiation can occur in the absence of B cells (Choi *et al*, 2011). DCs were an obvious next candidate. T-cell accumulation in B-cell follicles is regulated by DCs and is independent of B-cell activation (Fillatreau & Gray, 2003). A previous study performed using mice with a conditional deletion of the major TLR signaling adaptor MyD88, which is strictly required for TLR9 signaling in distinct cell types, found that intrinsic MyD88 signaling was required in DCs, but not in B cells, for the enhancing effect of CpG on antibody production in response to immunization with protein antigens. Although the formation of T_FH_ cells was not examined in that study, its conclusions are in agreement with the notion that it is intrinsic TLR signaling in DCs, rather than in B cells, that drives the increased formation of T_FH_ cells in response to CpG after immunization with protein antigens. Consistent with this, mice in which only CD11c^+^ DCs expressed MHC-II could generate T_FH_ cells (Goenka *et al*, 2011). Chakarov and Fazilleau took these data further by demonstrating the central role played by monocyte-derived DCs in the potentiating effect of CpG on the T_FH_-cell response. Using an elegant mixed bone marrow chimera system, they could show that intrinsic TLR9 signaling in CD11c^+^ DCs was driving the increased T_FH_-cell formation upon immunization with CpG. Remarkably, they then demonstrated that the elimination of monocyte-derived DCs was sufficient to entirely abolish the enhancing effect of CpG on T_FH_-cell accumulation. In contrast, eliminating monocyte-derived DCs had no impact on the T_FH_-cell response induced in the absence of CpG. CpG therefore stimulates the T_FH_-cell response by recruiting a pathway complementary but independent of the mechanism otherwise driving T_FH_-cell development. In line with this, the production of IL-6 was required for the T_FH_-cell-promoting effect of monocyte-derived DCs, while it was facultative for the induction of T_FH_ cell by conventional DCs. Monocyte-derived DCs required the expression of MHC-II to promote this effect, suggesting that they interacted with antigen-reactive T cells directly, rather than indirectly by modulating another subset of DCs. Monocyte DC-derived IL-6 could promote T_FH_-cell differentiation by stimulating the expression of Bcl-6 and ICOS in T cells, which are both essential for their engagement along the T_FH_-cell differentiation pathway. Depending on the immunization protocol, the formation of T_FH_ cell might therefore exhibit different requirement for IL-6 according to the respective contribution of conventional versus monocyte-derived DCs. The mechanisms involved in the promotion of T_FH_ cell by conventional DCs, and the identity of the relevant conventional DC subset(s), remain to be identified.

monocyte-derived DCs as booster of the T_FH_-cell response suggests that it could be possible to boost the protective value of vaccines

It is remarkable that CpG stimulated an increase in the T_FH_-cell response without augmenting the number of antigen-reactive CD4^+^ T cells, or altering the kinetics of this response in the draining lymphoid organ. Thus, the inclusion of CpG to the immunization cocktail did not lead to the recruitment of additional naïve T-cell precursors into the T-cell response, but rather influenced the differentiation course of primed T cells. Such effect of monocyte-derived DCs probably intercepted the trajectory of primed T cells during a time window between their priming by conventional DC and their irreversible engagement along a distinct differentiation pathway. Alternatively, monocyte DCs might have been the first antigen-presenting cells to interact with naïve T cells, preparing them for a subsequent interaction with conventional DCs that would lead to their differentiation into T_FH_ cells. This second possibility seems less likely because monocyte-derived DCs did not augment the global magnitude of the antigen-reactive T-cell response. In both cases, the effect of monocyte-derived DCs would take place early after immunization, in line with the observation that resection of the site of CpG deposition 1 h after immunization did not abrogate the augmentation of the T_FH_-cell response. The notion that T_FH_-cell commitment can start early is supported by the observation that Bcl-6 expression can be detectable in antigen-specific CD4^+^ T cells already during DC priming within 2 days after immune challenge (Choi *et al*, 2011). At this stage of the immune response, a large fraction of the primed T cells is certainly uncommitted. In fact, some activated T cells might persist in an unpolarized state throughout the immune response. The monocyte-derived DCs might create a microenvironment promoting the engagement of such ‘undecided’ T cells into the T_FH_-cell taskforce. An alternative possibility is that CpG induced a T_FH_-cell profile in cells that were about to initiate their engagement in another differentiation pathway. In this case, CpG would promote T_FH_-cell formation at the expense of other subsets of polarized T helper cells, which could be attractive to promote humoral immunity while limiting potentially unwanted inflammatory T-cell responses. Such shift in the T-cell subset balance could involve an inhibitory effect of Bcl-6, the master transcription factor of T_FH_ cells, on the expression of Blimp-1, which contributes to the formation of other T effector subsets. Further studies are required to distinguish between these possibilities and to delineate when monocyte-derived DCs and conventional DCs, respectively, interact with antigen-reactive T cells, which might precise the various DC-T-cell choreographies leading to T_FH_-cell formation. In any case, the T cells induced into T_FH_ cells under the influence of CpG seem functional because their accumulation was linked to a concomitant augmentation of the adaptive humoral response.

intrinsic TLR9 signaling in CD11c^+^ DCs was driving the increased T_FH_-cell formation upon immunization with CpG

The identification of monocyte-derived DCs as booster of the T_FH_-cell response suggests that it could be possible to boost the protective value of vaccines by targeting these cells. A human equivalent of monocyte-derived DCs was recently identified (Segura *et al*, 2013). Moreover, Chakarov and Fazilleau showed that antigens enclosed in vesicles specifically internalized by monocyte-derived DCs efficiently promoted humoral immunity. However, they did not directly compare side-by-side soluble antigen delivery versus antigen enclosed in beads for their capacities to promote T_FH_-cell formation and long-lasting humoral immunity so it is not yet possible to conclude whether such mode of antigen delivery actually presents a specific advantage in terms of vaccine efficacy. An important point to keep in mind with regard to the targeting of such DCs is, however, that DCs derived from Ly6C^+^ blood monocytes are very plastic and also able to promote T_H_1- as well as T_H_2-cell responses depending on the inflammatory environment. Moreover, the human monocyte-derived DCs recently identified in the synovial fluid of rheumatoid arthritis patients and ascites fluid from cancer patients were potent inducers of T_H_17-cell differentiation *in vitro* (Segura *et al*, 2013). The various effects of monocyte-derived DCs are intriguing because the differentiation of T cells into T_FH_ cell versus other effector T helper cell subsets seems to be regulated antagonistically by Bcl-6 and Blimp-1, respectively (Johnston *et al*, 2009). There is currently little understanding of the implications of monocyte-derived DCs in the promotion of such diverse types of T-cell responses. However, this knowledge would be valuable because the induction of DCs capable of promoting inflammatory T-cell responses might increase the risk of provoking unwanted side effects upon vaccination. It would therefore be useful to understand what determines whether monocyte-derived DCs will induce T_FH_-, T_H_1-, T_H_2-, or T_H_17-cell differentiation upon interaction with uncommitted T cells, in order to develop tools that selectively promote their positive effect on humoral immunity. A possibility could be that they preferentially trigger T_FH_ cell versus other types of T helper cell responses depending on the microenvironment where the response takes place. For instance, they could stimulate T_FH_-cell formation when located in the inter-follicular zone, where T_FH_-cell priming has been observed. The possibility of labeling these cells using their phagocytic activity should facilitate the elucidation of their migration pattern in different contexts and could permit their targeting with small RNA or pharmacological agents to modulate their function.

In sum, the study by Chakarov and Fazilleau shows that CpG acts on a flexible component of the immune system, that is, monocyte-derived DCs to promote, when primed CD4^+^ T cells have an uncommitted phenotype, their differentiation along the T_FH_-cell pathway. This work complements recent studies that underscored how the structural properties of the antigen determined the mechanism contributing to humoral immunity (Geeraedts *et al*, 2008; Hou *et al*, 2011). Such ongoing progress in our understanding of the modes of action of adjuvant shall allow the development of safer and more efficient vaccines.

## References

[b1] Amanna IJ, Carlson NE, Slifka MK (2007). Duration of humoral immunity to common viral and vaccine antigens. N Engl J Med.

[b2] Chakarov S, Fazilleau N (2014). Monocyte-derived dendritic cells promote T follicular helper cell differentiation. EMBO Mol Med.

[b3] Choi YS, Kageyama R, Eto D, Escobar TC, Johnston RJ, Monticelli L, Lao C, Crotty S (2011). ICOS receptor instructs T follicular helper cell versus effector cell differentiation via induction of the transcriptional repressor Bcl6. Immunity.

[b4] Cooper CL, Davis HL, Angel JB, Morris ML, Elfer SM, Seguin I, Krieg AM, Cameron DW (2005). CPG 7909 adjuvant improves hepatitis B virus vaccine seroprotection in antiretroviral-treated HIV-infected adults. AIDS.

[b5] Crotty S (2011). Follicular helper CD4 T cells (TFH). Annu Rev Immunol.

[b6] Fillatreau S, Gray D (2003). T cell accumulation in B cell follicles is regulated by dendritic cells and is independent of B cell activation. J Exp Med.

[b7] Geeraedts F, Goutagny N, Hornung V, Severa M, de Haan A, Pool J, Wilschut J, Fitzgerald KA, Huckriede A (2008). Superior immunogenicity of inactivated whole virus H5N1 influenza vaccine is primarily controlled by Toll-like receptor signalling. PLoS Pathog.

[b8] Goenka R, Barnett LG, Silver JS, O'Neill PJ, Hunter CA, Cancro MP, Laufer TM (2011). Cutting edge: dendritic cell-restricted antigen presentation initiates the follicular helper T cell program but cannot complete ultimate effector differentiation. J Immunol.

[b9] Hou B, Saudan P, Ott G, Wheeler ML, Ji M, Kuzmich L, Lee LM, Coffman RL, Bachmann MF, DeFranco AL (2011). Selective utilization of Toll-like receptor and MyD88 signaling in B cells for enhancement of the antiviral germinal center response. Immunity.

[b10] Johnston RJ, Poholek AC, DiToro D, Yusuf I, Eto D, Barnett B, Dent AL, Craft J, Crotty S (2009). Bcl6 and Blimp-1 are reciprocal and antagonistic regulators of T follicular helper cell differentiation. Science.

[b11] Segura E, Touzot M, Bohineust A, Cappuccio A, Chiocchia G, Hosmalin A, Dalod M, Soumelis V, Amigorena S (2013). Human inflammatory dendritic cells induce Th17 cell differentiation. Immunity.

[b12] Weber JP, Fuhrmann F, Hutloff A (2012). T-follicular helper cells survive as long-term memory cells. Eur J Immunol.

